# Supplementary material for: Reorganization of nurse scheduling reduces the risk of healthcare associated infections
Source: Sci Rep. 2021 Apr 1;11:7393. doi: 10.1038/s41598-021-86637-w (PMC8016903; doi:10.1038/s41598-021-86637-w)
Supplement: Supplementary file 1 — Supplementary Information. [file 41598_2021_86637_MOESM1_ESM.pdf]

SUPPLEMENTARY INFORMATION

Reorganization of nurse scheduling reduces the risk of healthcare associated infections

Eugenio Valdano[1], Chiara Poletto[1], Pierre-Yves Boëlle[1], Vittoria Colizza[1]

[1]*INSERM, Sorbonne Université, Institut Pierre Louis d'Epidémiologie et de Santé Publique (IPLESP), 75012, Paris, France*

1. Contact data collection

Staff and patients of the geriatric unit were equipped with wearable RFID (Radio-Frequency Identification) devices in the form of badges to wear on their chest. Devices could identify one another via the exchange of radio packets, and signal contact between them. Intensity of radio packages was tuned to allow data exchange up to a distance 1-to-1.5 meters. Also, the frequency used is shielded by water in the human body. This ensured that devices signaled contact between them only in case of close-range, face-to-face proximity. Devices also communicated to fixed antennas whose range covered the whole ward. This experimental infrastructure had 99% probability of detecting a close-proximity event lasting 20 seconds. As such, contact initiation was defined as the exchange of at least one radio-frequency packet over a time span of 20 seconds. Contacts ended when, in a time span of 20 seconds, no radio-frequency package was exchanged. The technical details of the data collection are available from the data provider website [1], and Ref. [2].

2. Infection propagator approach to evaluate the HAI risk

We represent the time-evolution of contacts in terms of a temporal network with adjacency matrices A_t, with t running on the 1-hour time steps. The entry i, j of A_t is equal to one if nodes i, j establish a contact during time step t, zero otherwise. We model the spread of the pathogen using a Susceptible – Colonized – Susceptible model. A colonized node in the network transmits the pathogen to a connected node with probability λ (transmissibility) at each time step. It also clears the pathogen with a probability μ at each timestep. μ^{-1} is then the average carriage period. There exists a critical value of transmissibility λ_c – called epidemic threshold – that determines the global behavior of the outbreak. If transmissibility is higher than the epidemic threshold ($\lambda > \lambda_c$), introducing the pathogen into the hospital ward is likely to cause a large-scale outbreak. Instead, if transmissibility is lower than the epidemic threshold ($\lambda < \lambda_c$), the outbreak is likely to affect few individuals. Therefore, computing changes in the epidemic threshold is a a synthetic and easy-to-interpret way to weigh the impact of any policy, on the

vulnerability of the ward to the pathogen considered. If the epidemic threshold increases following intervention, the ward becomes more resilient to pathogen introduction. Oppositely, if the epidemic threshold goes down, the ward becomes more prone to large-scale outbreaks. This is the rationale behind our definition of HAI risk reduction: $(\lambda_c^{INT} - \lambda_c^{EMP})/\lambda_c^{EMP}$. In order to compute it, we need to compute the epidemic threshold before and after intervention. To that end, we employ the infection propagator approach [3-5], which can compute the epidemic threshold on any arbitrary temporal network, for the spreading model used here. The infection propagator is the following matrix:

$$P(\lambda, \mu) = \prod_t (1 - \mu + \lambda A_t).$$

It contains both the time-evolving structure of the contact network (A_t), and the parameters of the spreading model (λ, μ), and measures the chains of infection between individuals along which the pathogen can spread. We prove in [3-5] that the epidemic threshold is the smallest value of λ for which the largest eigenvalue of P equals one.

We provide a Python library to compute the epidemic threshold of any empirical temporal network in the following repository: https://github.com/eugenio-valdano/threshold

3. Implementation of switch and reassignment of nurses' tasks and minimization of the potential

We minimize the potential using the Metropolis algorithm. It is an iterative process based on the following steps.

Version for S_{+1}, S_{-1}:

1) Choose two nurses ($i \neq j$), and one time step (t);
2) If neither nurse is active during t, go to 1);

3) Swap tasks between i, j during t;

4) If the swap breaks the S constraint, go to 1);

5) Compute the potential;

6) If the swap decreases the potential, accept the swap. If the swap increases the potential, accept it with probability $e^{-\Delta V}$, where ΔV is the change in potential due to the swap;

7) Only if the swap is accepted, update nurses' task assignments, and potential;

8) Go to 1).

Version for $\mathrm{W}S_{+1}, WS_{-1}$:

1) Choose two nurses $(i \neq j)$;

2) Choose two time steps (t, s), so that i is active during t, and not active during s, and j is active during s, and not active during t. If this is not possible, go to 1);

3) Swap tasks between i, j during both t, s;

4) If the swap breaks the S constraint, go to 1);

5) Compute the potential;

6) If the swap decreases the potential, accept the swap. If the swap increases the potential, accept it with probability $e^{-\Delta V}$, where ΔV is the change in potential due to the swap;

7) Only if the swap is accepted, update nurses' task assignments, and potential;

8) Go to 1).

REFERENCES

1. www.sociopatterns.org. Accessed March 2021.

2. Vanhems P, Barrat A, Cattuto C, Pinton J-F, Khanafer N, Régis C, Kim B, Comte B, Voirin N. Estimating Potential Infection Transmission Routes in Hospital Wards Using Wearable Proximity Sensors. PLOS One. 2013 8(9): e73970.

3. Valdano E, Ferreri L, Poletto C, Colizza V. Analytical Computation of the Epidemic Threshold on Temporal Networks. Phys Rev X. 2015 Apr 8;5(2):021005.

4. Valdano E, Poletto C, Colizza V. Infection propagator approach to compute epidemic thresholds on temporal networks: impact of immunity and of limited temporal resolution. Eur Phys J B [Internet]. 2015 Dec [cited 2016 Jan 3];88(12). Available from: http://link.springer.com/10.1140/epjb/e2015-60620-5

5. Darbon A, Colombi D, Valdano E, Savini L, Giovannini A, Colizza V. Disease persistence on temporal contact networks accounting for heterogeneous infectious periods. R Soc Open Sci. 2019 Jan;6(1):181404.
